# The Amyloid Precursor Protein Controls PIKfyve Function

**DOI:** 10.1371/journal.pone.0130485

**Published:** 2015-06-30

**Authors:** Zita Balklava, Christian Niehage, Heather Currinn, Laura Mellor, Benjamin Guscott, Gino Poulin, Bernard Hoflack, Thomas Wassmer

**Affiliations:** 1 Aston University, School of Life and Health Sciences, Aston Triangle, Birmingham, B4 7ET, United Kingdom; 2 Biotechnologisches Zentrum, TU-Dresden, Tatzberg 47–49, 01307 Dresden, Germany; 3 University of Manchester, Michael Smith Building, Oxford Road, Manchester, M13 9PT, United Kingdom; University of S. Florida College of Medicine, UNITED STATES

## Abstract

While the Amyloid Precursor Protein (APP) plays a central role in Alzheimer’s disease, its cellular function still remains largely unclear. It was our goal to establish APP function which will provide insights into APP's implication in Alzheimer's disease. Using our recently developed proteo-liposome assay we established the interactome of APP's intracellular domain (known as AICD), thereby identifying novel APP interactors that provide mechanistic insights into APP function. By combining biochemical, cell biological and genetic approaches we validated the functional significance of one of these novel interactors. Here we show that APP binds the PIKfyve complex, an essential kinase for the synthesis of the endosomal phosphoinositide phosphatidylinositol-3,5-bisphosphate. This signalling lipid plays a crucial role in endosomal homeostasis and receptor sorting. Loss of PIKfyve function by mutation causes profound neurodegeneration in mammals. Using *C*. *elegans* genetics we demonstrate that APP functionally cooperates with PIKfyve *in vivo*. This regulation is required for maintaining endosomal and neuronal function. Our findings establish an unexpected role for APP in the regulation of endosomal phosphoinositide metabolism with dramatic consequences for endosomal biology and important implications for our understanding of Alzheimer's disease.

## Introduction

The transmembrane protein Amyloid Precursor Protein (APP, NCBI: NM_000484) is the central player in Alzheimer’s disease, as patients with mutations in the APP gene develop a familial form of Alzheimer’s disease [1, 2]. APP is processed by beta and gamma secretases into three main fragments: the beta amyloid peptide (approximately 40 amino acids in length) which is released from neurons, the extracellular domain of APP (sAPPb) and the intracellular domain termed AICD. Cleavage of APP by the gamma secretase complex is instrumental in Alzheimer’s disease as documented by the identification of familial Alzheimer’s disease mutations in gamma secretase complex subunits (reviewed in [3]).

Despite the importance of APP in Alzheimer's disease it still remains unclear what exactly the physiological function of this molecule is. Due to its prototypical structure APP was suggested to engage in cell signalling through proteolytic cleavage by beta and gamma secretases in a fashion highly similar to that of the Notch signalling receptor [4]. Indeed, analogous to Notch signalling, gamma secretase mediated release of AICD from the membrane has been suggested to mediate signalling to the nucleus (reviewed in [5]).

Arguing for a role of APP in cell signalling but proposing an entirely different mechanism the *Drosophila* homologue of APP, APPL, was shown to be involved in Wnt/planar cell polarity signalling via the Abelson kinase [6]. Furthermore, APPL was shown to regulate axonal outgrowth and neuronal regeneration after brain injury [7]. Numerous additional roles have been ascribed to APP (reviewed in [8]), among them an involvement in synapse organisation [9]. Despite these observations no coherent model of APP function has emerged.

APP, as a type-I transmembrane protein, is known to cycle between the plasma membrane, endosomes and the trans-Golgi network [10]. It was shown to be sorted from the limiting membrane of sorting endosomes for recycling to the trans-Golgi network requiring a trafficking complex known as the Retromer [11]. How the trafficking of APP and, more specifically, its endosomal sorting is linked to its proposed function in cell signalling remains unclear.

Endosomal sorting requires the endosomal phospholipid phosphatidylinositol-3-phosphate (PI(3)P). This phosphoinositide provides membrane attachment for numerous endosomal sorting complexes, Retromer sorting nexins being a well characterised group [12–14]. Additionally, the PI(3)P derived phosphatidylinositol-3,5-bisphosphate (PI(3,5)P_2_) is required for Retromer dependent transport [15]. This lipid is produced by the PI(3)P-specific PIKfyve kinase (also known as Fab1) that produces PI(3,5)P_2_. How exactly PI(3,5)P_2_ controls endosome-to-TGN transport is not currently clear. In addition to regulating endosome-to-TGN transport, PI(3,5)P_2_ controls endosomal morphology and function [16, 17]. PI(3,5)P_2_, regulates calcium permeability of endosomes via the TRP channel TRPML1 [18]. This is essential for endosomal homeostasis and the degradative capability of the endosomal system. Defects in TRPML1 lead to mucolipidosis type IV, a form of lysosomal storage disease that causes profound neurodegeneration [19]. The most visible consequence of loss of the PI(3,5)P_2_-producing enzyme PIKfyve, induced either by RNAi suppression [15], expression of a kinase-dead mutant [20], mutation [21] or pharmacological inhibition [22] is the extreme swelling of late endosomes to form vacuoles.

PIKfyve functions as part of a conserved protein complex together with its co-activators Vac14 and Fig 4, (also known as ArPIKfyve and Sac3, respectively) [23, 24]. Crucially, mouse Fig 4 loss-of-function mutations or Vac14 knock-out mutants not only reduce PI(3,5)P_2_ levels but also lead to profound neurodegeneration, resulting in perinatal death of the animals [21, 25]. Furthermore, mutations identified in the human Fig 4 gene lead to neurodegeneration in humans including Charcot-Marie-Tooth syndrome and Amyotrophic Lateral Sclerosis [21, 26].

In this study we demonstrate that the key player in Alzheimer's disease, APP, interacts with the PIKfyve complex and regulates the PIKfyve pathway in *C*. *elegans*, establishing an entirely novel role for APP. The unexpected link between APP and endosomal phosphoinositide metabolism may suggest a novel and surprising mechanism for neurodegeneration in Alzheimer's disease.

## Results

### APP binds Vac14 of the mammalian PIKfyve complex

For understanding the elusive physiological role of APP we studied the interactome of APP's intracellular domain (termed AICD) using an *in-vitro* reconstitution system that we have recently established [27]. In this system AICD was coupled to the surface of liposomes to mimic the organisation of its intracellular domain in the native APP together with a membrane context [28]. These so-called 'proteo-liposomes' were then used to recruit interaction partners followed by isolation and identification by mass spectrometry [27–29].

**Fig 1:**
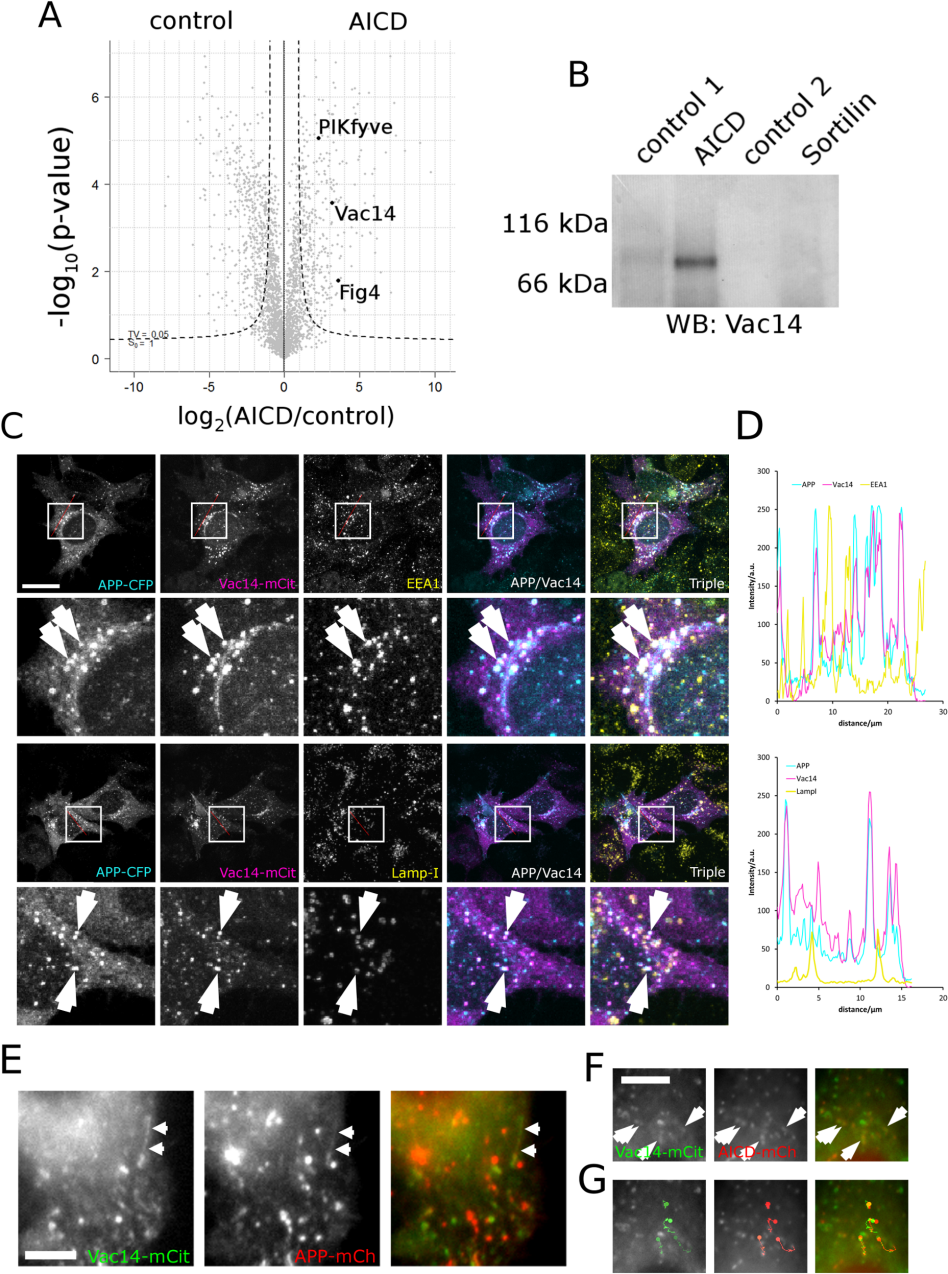
Vac14 associates with the intracellular domain of APP (AICD) biochemically and in HeLa cells. (A) Proteo-liposome recruitment analysed by mass spectrometry allowed the identification of the intracellular interactome of APP. A Vulcan plot showing that PIKfyve, Vac14 and Fig 4 of the mammalian PIKfyve complex were significantly enriched in AICD proteo-liposomes (right quadrant) compared to controls (left). The dashed line indicates the 0.05 significance threshold. (B) Western blotting confirmed the enrichment of Vac14 by AICD presenting proteo-liposomes while the intracellular domain of the none-related receptor Sortilin and two additional controls (a non-related control peptide designated as 'control 1' or coupled cysteine 'control 2'–both described in [27]) failed to bind Vac14, showing that Vac14 specifically associates with the intracellular domain of APP. (C) APP expressed as a C-terminal CFP fusion displayed strong colocalisation with Vac14-mCit in HeLa cells. Limited colocalisation of APP and Vac14 could be observed on early endosomes (labelled with EEA1) and late endosomes/lysosomes labelled with LampI, as indicated by arrows on the insets. Bar, 20 μm. (D) Line scans (indicated by the red line in (C) demonstrated strong colocalisation between APP and Vac14. (E) In live-cell imaging APP fused to mCherry displayed co-movement with Vac14-mCit positive vesicles and tubular carriers (arrows) that track through the cytoplasm. (F, G) (S1 Video). AICD fused to mCherry also colocalised with Vac14-mCit in live cell imaging (arrows) (S2 Video). (G) Four individual vesicles positive for both AICD and Vac14 were tracked and the first image of the sequence overlayed with the traces to illustrate comigration of AICD and Vac14. Note that in live cell imaging due to the delay caused by the change of filters on fast moving vesicles the staining in the red and green channels are slightly displaced. Bar, 5μm.

Combining AICD proteo-liposomes with label-free mass spectrometry [30] we established the interactome of APP's intracellular domain and, surprisingly, found all three subunits of the mammalian PIKfyve complex, namely PIKfyve (NCBI: NM_015040), Vac14 (NCBI: NM_018052) and Fig 4 (NCBI: NM_014845) to associate with APP's intracellular domain (Fig 1). Given that inactivation of the PIKfyve complex leads to neurodegeneration and that APP plays a key role in Alzheimer's disease this finding prompted us to investigate this interaction in more detail.

We confirmed the interaction between the intracellular domain of APP and Vac14 and the PIKfyve complex by analysing the protein recruitments using Western blotting, showing that Vac14 of the PIKfyve complex binds specifically to AICD-presenting proteo-liposomes while no significant amounts of Vac14 were found in any of three different, negative controls (Fig 1).

### APP co-localises and co-migrates with Vac14 in mammalian cells

When analysing the steady state localisation of APP and Vac14 in HeLa cells by co-expressing APP-CFP and Vac14-mCIT we found them to display strong colocalisation on vesicles (Additional line scans are presented in S1 Fig). Some of these were positive for EEA1 indicating early endosomes while some were LampI positive late endosomes (Fig 1). The dynamic association of APP with Vac14 was also observed in live-cell imaging where APP co-localises and co-migrates with Vac14 on tubules and vesicles moving through the cytoplasm (Fig 1). Additionally, we detected significant colocalisation on mobile vesicles when expressing only the intracellular domain of APP, AICD, fused to mCherry together with Vac14 (Fig 1). This is interesting given that AICD, lacking a transmembrane domain, is expected to be cytosolic. However, these data show that a portion of AICD is clearly membrane associated and colocalises with Vac14. These data are consistent with the APP/Vac14 interaction established in Fig 1 and suggest that APP associates with the PIKfyve complex in cells via its intracellular domain.

### Analysis of APP and PIKfyve function in *C*. *elegans*

What is the function of the protein-protein interaction between APP and the PIKfyve complex *in-vivo*? Currently it is not clear how PIKfyve function is regulated in multicellular organisms. The fact that APP binds the PIKfyve activator Vac14 raised the possibility that APP may control PIKfyve activity. For testing this hypothesis we used the genetic model organism *Caenorhabditis elegans*. The significant advantage of *C*. *elegans* is that only one orthologue of APP exists (named *apl-1*, NCBI: NM_076469). In contrast, in mammals the APP-like gene family consists of APP, APLP-1 and APLP-2. Mouse genetics clearly demonstrated a high level of functional redundancy between these three genes, considerably complicating the analysis of APP gene function in mammals [31].

Both APL-1 and PIKfyve (PPK-3 in *C*. *elegans*, NCBI: NM_077754) are essential for worm development and viability [32, 33]. The most prominent phenotype of *apl-1* null alleles is developmental arrest during larval stages caused by a loose cuticle. This lethal phenotype is rescued by the expression of the soluble, extracellular domain of APL-1 [32]. Whether APL-1 has additional functions that are masked by the severity of the cuticle phenotype remains unclear.

The PIKfyve orthologue PPK-3 is also essential and required for embryogenesis [33]. Additionally, PPK-3 plays an important role in endosomal homeostasis. Partial loss-of-function alleles (hypomorphs) lead to a drop in PI(3,5)P_2_ levels and cause the formation of large vacuoles in hypodermal and intestinal cells, mirroring closely the defects induced by loss of mammalian PIKfyve [33]. The *vacl-14* gene (NCBI: NM_059814), the *C*. *elegans* orthologue of mammalian Vac14, has not been characterised in *C*. *elegans* to date.

First, we wanted to test whether in *C*. *elegans* the putative Vac14 orthologue VACL-14 plays a similar role as Vac14 in the mammalian complex and is indeed required for the activation of PPK-3. We tested this by creating the *ppk-3(n2835); vacl-14(ok1877)* double mutant which showed synthetic lethality, confirming that in *C*. *elegans* VACL-14 is necessary for full PPK-3 function and suggesting that the *C*. *elegans* complex functions similarly as the human complex.

### Truncation of APL-1 leads to vacuole formation

To test whether APL-1 and the PPK-3 complex share a common function we analysed the phenotypes of *apl-1*, *ppk-3* and *vacl-14* mutant alleles. For APL-1 we chose to study the *yn5* deletion in which the 3'-end of the gene encoding the transmembrane domain and intracellular domain are deleted [32]. The *yn5* allele thus represents a powerful tool for characterising the role of the cytoplasmic domain of APL-1 without additional, deleterious effects caused by lack of the extracellular domain [32]. This approach allowed us to characterise phenotypes that are masked by the lethality of *apl-1* null mutants. According to our working hypothesis APL-1 binds the PPK-3 complex via its intracellular domain and controls its activity. Loss of the intracellular domain of APL-1 will prevent it from binding to the PPK-3 complex and disrupt PPK-3 regulation. By consequence, APL-1 mutants should have a similar phenotype as PPK-3 loss-of-function mutations.

**Fig 2:**
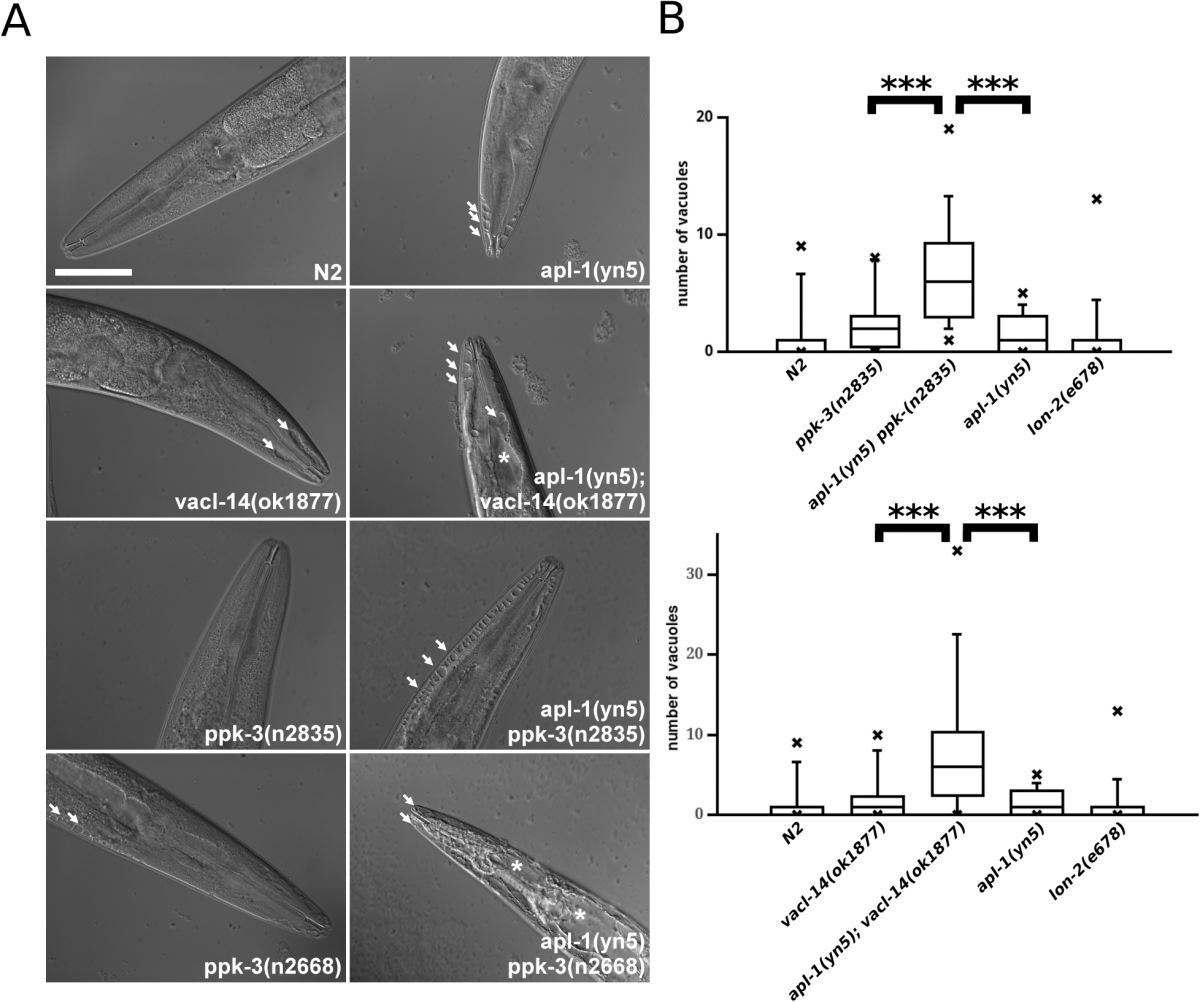
APL-1 interacts genetically with the PIKfyve complex genes vacl-14 and ppk-3. (A) Mutations in apl-1, ppk-3 and vacl-14 led to the formation of vacuoles in the C. elegans intestine and hypoderm (indicated by arrows) apparent in the anterior tips of the worms as observed by Differential Interference Contrast (DIC) microscopy. Combination of apl-1, ppk-3 and vacl-14 mutations in double mutant worms strongly enhanced this phenotype (* indicate very large vacuoles), particularly evident in the apl-1(yn5) ppk-3(n2668) double mutant. (B) Box plots demonstrated that the number of vacuoles in apl-1/ppk-3 and apl-1/vacl-14 double mutants is significantly increased compared to the single mutants (Wilcoxon rank test, p<0.01, n≥20 per strain), demonstrating that APL-1 functionally interacts with the PPK-3 complex. This showed that the C-terminal domain of APL-1 is necessary for suppressing vacuole formation induced by loss of PPK-3 and VACL-14 activity. Bar, 50 μm.

Reduced PPK-3 activity by partial loss of function mutations in *ppk-3* or deletion of its activator VACL-14 led to the formation of large vacuoles that are most easily visible in hypodermal cells ([33] and Fig 2). Interestingly, when analysing the *apl-1(yn5)* mutant we observed hypodermal vacuolation similar to that of *ppk-3* and *vacl-14* mutants. This showed that the *apl-1(yn5)* mutant phenocopies the partial loss of PPK-3 function (Fig 2 and [32]).

### APL-1 functionally cooperates with PPK-3

While this observation suggests that APL-1 and PPK-3 may share a common function it is not clear whether they act in the same pathway. To test this we analysed the epistasis of the *apl-1* and *ppk-3* genes. For this we combined the *apl-1(yn5)* mutation with hypomorphic alleles of *ppk-3*. For reducing PPK-3 activity two hypomorphic *ppk-3* alleles were used. *ppk-3(n2668)*, introducing a point mutation in the catalytic loop, significantly reduced PPK-3 activity [33]. A weaker allele, *ppk-3(n2835)*, leads to the formation of a truncated PPK-3 lacking the C-terminal eleven amino acids [33]. Additionally, we utilised the *vacl-14(ok1877)* deletion mutant. Using these mutants we created double mutants in the *apl-1*, *ppk-3* and *vacl-14* genes and compared their phenotypes. In all three double mutants [*apl-1(yn5); vacl-14(ok1877)*, *apl-1(yn5) ppk-3(n2835) and apl-1(yn5) ppk-3(n2668)*] the accumulation of strongly swollen vacuoles was evident (Fig 2 and S2 Fig). These proved to be significantly more numerous compared to single mutants or control animals (Fig 2), showing that truncation of APL-1 considerably enhanced the defects caused by reduced PPK-3 activity. This was particularly evident in the *apl-1(yn5) ppk-3(n2668)* double mutant containing the stronger *ppk-3* mutant allele. These animals were filled with vacuoles along the entire length of the animal in both the hypoderm and intestine (Fig 2A and S2 Fig). The strength of the phenotypic effect of this double mutant precluded us from counting individual vacuoles, as large portions of the animals were filled with vacuoles. Moreover, these animals were unable to produce any viable offspring and the strain was non-viable. Thus the truncation mutation in *apl-1(yn5)* combined with the *ppk-3(n2668)* point mutation are synthetically lethal, demonstrating that APL-1 is required for PPK-3 function for suppressing vacuoles and maintaining viability.

For independent confirmation of the results obtained using the *apl-1(yn5)* mutant we used RNAi suppression of APL-1 and showed that knock-down of APL-1 led to the accumulation of huge vacuoles in the hypoderm and intestine of *ppk-3* mutant animals compared to wildtype controls (S3 Fig), mirroring the effect of truncating APL-1. These data show that it is indeed the loss of APL-1 that enhances loss of PPK-3 function.

**Fig 3:**
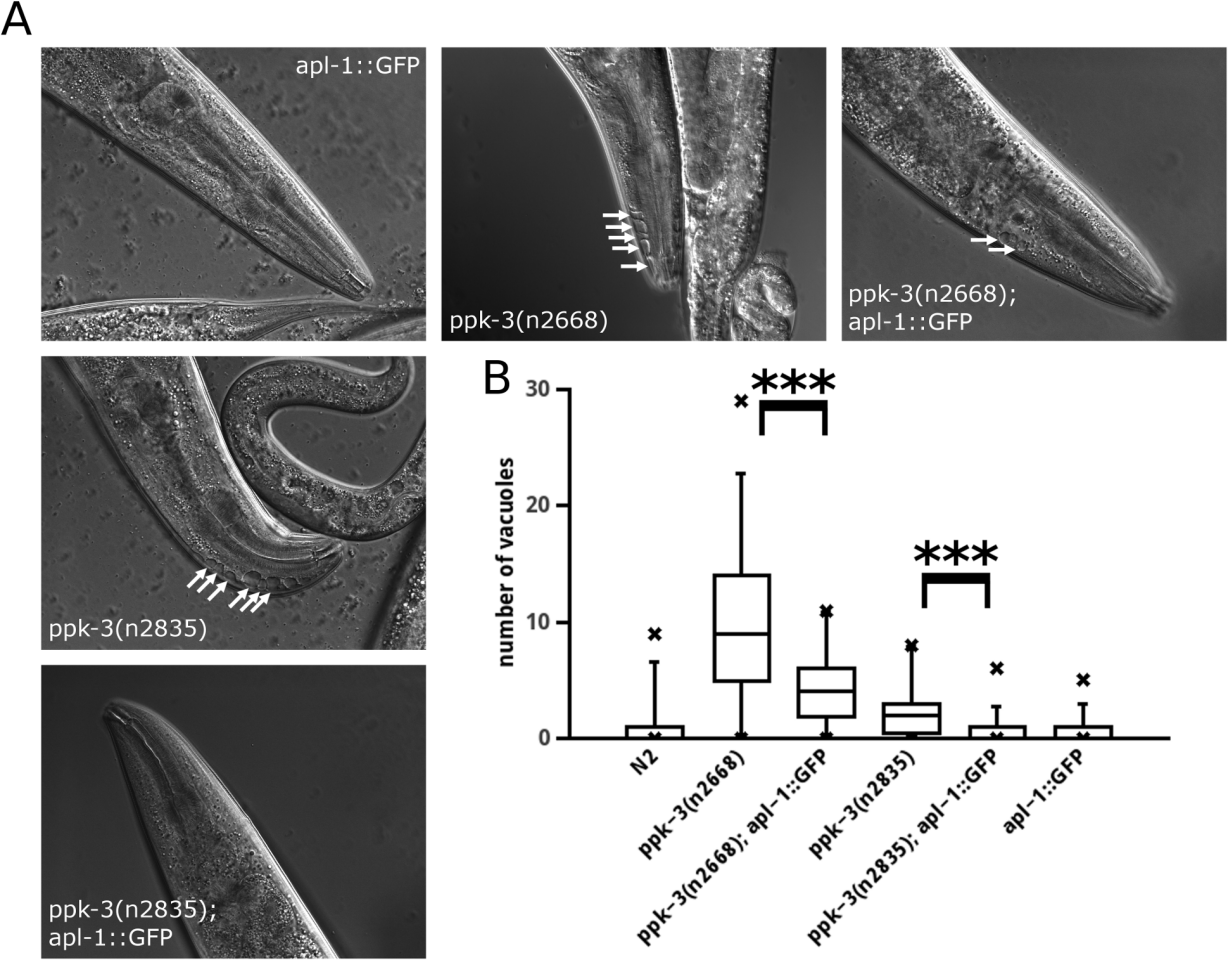
Overexpression of APL-1 reduces vacuolar pathology caused by hypomorphic PIKfyve complex mutants. (A, B) Overexpression of APL-1::GFP (established in [32]) significantly reduced the number of vacuoles in PPK-3 partial loss-of-function mutant animals (Wilcoxon rank test, p<0.01, n≥25 per strain). Note that in the case of the stronger hypomorphic ppk-3(n2668) mutant APL-1::GFP expression, while strongly ameliorating the phenotype, failed to fully rescue the vacuolar phenotype.

If loss of APL-1 exacerbates the effects of PPK-3 impairment it is reasonable to assume that overexpression of APL-1 may rescue the PPK-3 deficiency phenotype. We tested this by expressing APL-1::GFP (characterised in [32]) in both hypomorphic *ppk-3* mutant strains and measured the number of vacuoles. In Fig 3 it is shown that overexpression of APL-1::GFP significantly reduced the number of vacuoles in both *ppk-3* mutants. These data are compatible with APL-1 functioning within the PPK-3 pathway.

While APL-1 and PPK-3 appear to act in the same pathway it was not clear whether APL-1 acts upstream or downstream of PPK-3. Furthermore we have not fully excluded the possibility that APL-1 and PPK-3 share a common function but act in parallel pathways.

**Fig 4:**
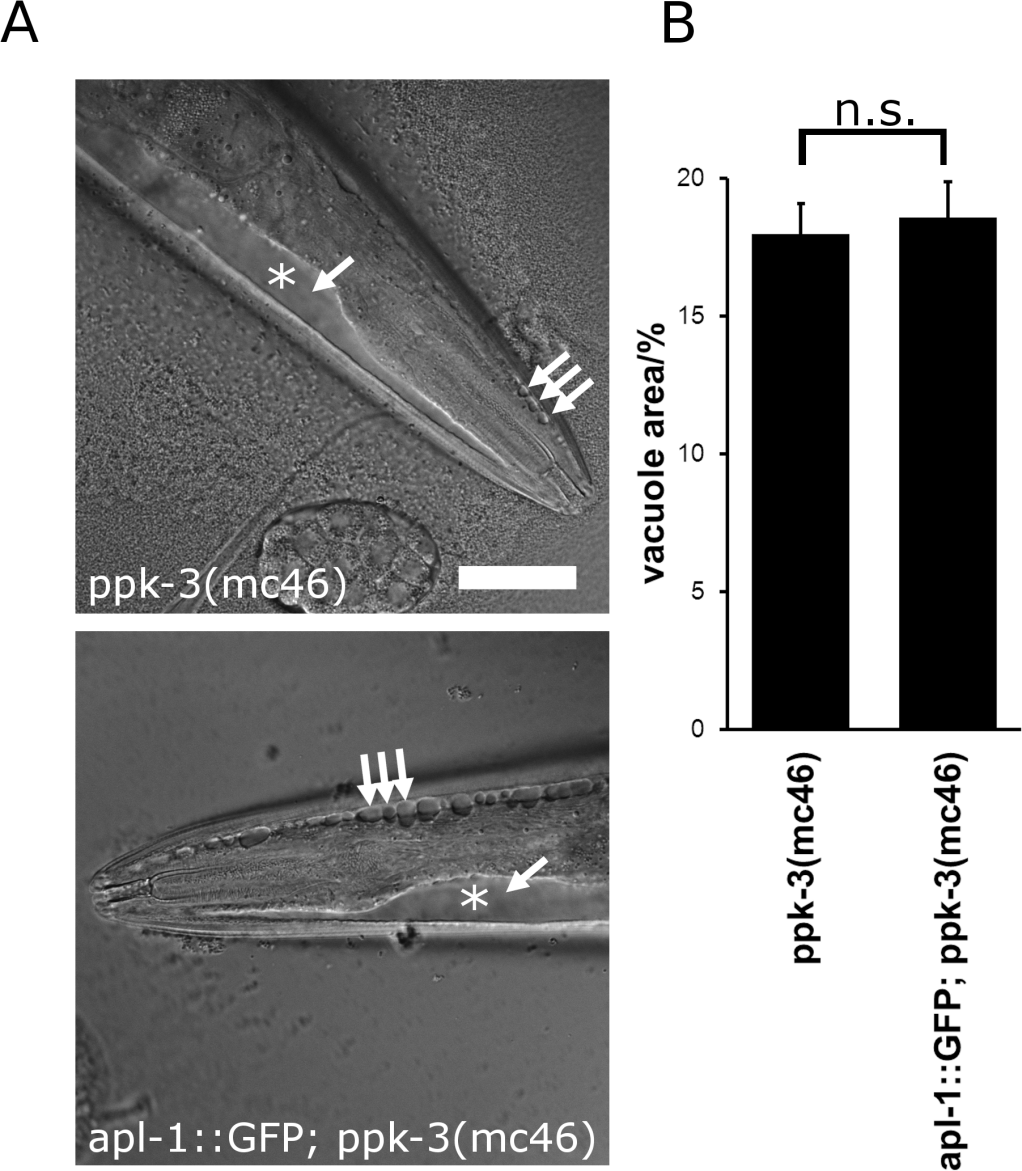
APL-1 overexpression, while able to rescue partial loss of PPK-3 function, failed to rescue the ppk-3(mc46) null allele. (A) Expression of APL-1::GFP failed to rescue lethality of ppk-3 null animals and failed to rescue vacuolation in apl-1::GFP; ppk-3(mc46) animals. Bar, 50μm. (B) Quantification of the relative vacuolated area in ppk-3(mc46) and apl-1::GFP; ppk-3(mc46) animals showed that APL-1 overexpression failed to rescue complete loss of PPK-3 function (n≥38, p = 0.73 (two-tailed t-test), suggesting that APL-1 functions upstream of PPK-3.

Overexpression of APL-1 ameliorated the partial loss of PPK-3 function induced by hypomorphic *ppk-3* alleles. If APL-1 acted downstream of PPK-3, overexpression of APL-1 should also ameliorate the phenotype caused by complete loss of PPK-3 function in a *ppk-3* null mutant. Similarly, if APL-1 functions in a pathway parallel to PIKfyve, overexpression of APL-1 should also rescue the phenotype of a *ppk-3* null allele. In contrast, overexpression of APL-1 should not result in a rescue if APL-1 functions upstream of PPK-3. We tested the hypothesis that APL-1 functions upstream of PPK-3 by overexpressing APL-1::GFP in the homozygous *ppk-3(mc46)* null mutant [33]. *ppk-3* null animals from heterozygous hermaphrodite mothers grow up to the adult stage but fail to produce any viable offspring. Overexpression of APL-1::GFP in hermaphrodites did not rescue lethality of their offspring. Additionally, we studied vacuolation in the anterior tip of young *apl-1*::*GFP; ppk-3(mc46)* adults by measuring the area of the anterior tip covered by vacuoles. This was necessary as the vacuolation phenotype in these animals was so pronounced that it was rarely possible to discriminate individual vacuoles because of their fused appearance (Fig 4). An average 17% of the anterior tip was covered by vacuoles in both the *ppk-3(mc46)* and the *apl-1*::*GFP; ppk-3(mc46)* animals, showing that APL-1 overexpression did not rescue the *ppk-3* null phenotype.

Taken together, APL-1::GFP expression is able to rescue the vacuolation phenotype of hypomorphic *ppk-3* alleles while it appears to be unable to rescue a *ppk-3* null allele, suggesting that APL-1 acts upstream of PPK-3. One caveat of this analysis is that vacuolation may not be a very sensitive measure of PPK-3 function which may not allow to detect subtle changes of PPK-3 activity. Thus we cannot fully rule out the possibility that APL-1 may act in a pathway parallel to PPK-3.

However, given that APP interacts with the PIKfyve complex in mammals (Fig 1) the most plausible explanation is that of APP/APL-1 binding to and activating PIKfyve/PPK-3.

### APP controls endo- and lysosomal homeostasis together with PIKfyve

**Fig 5:**
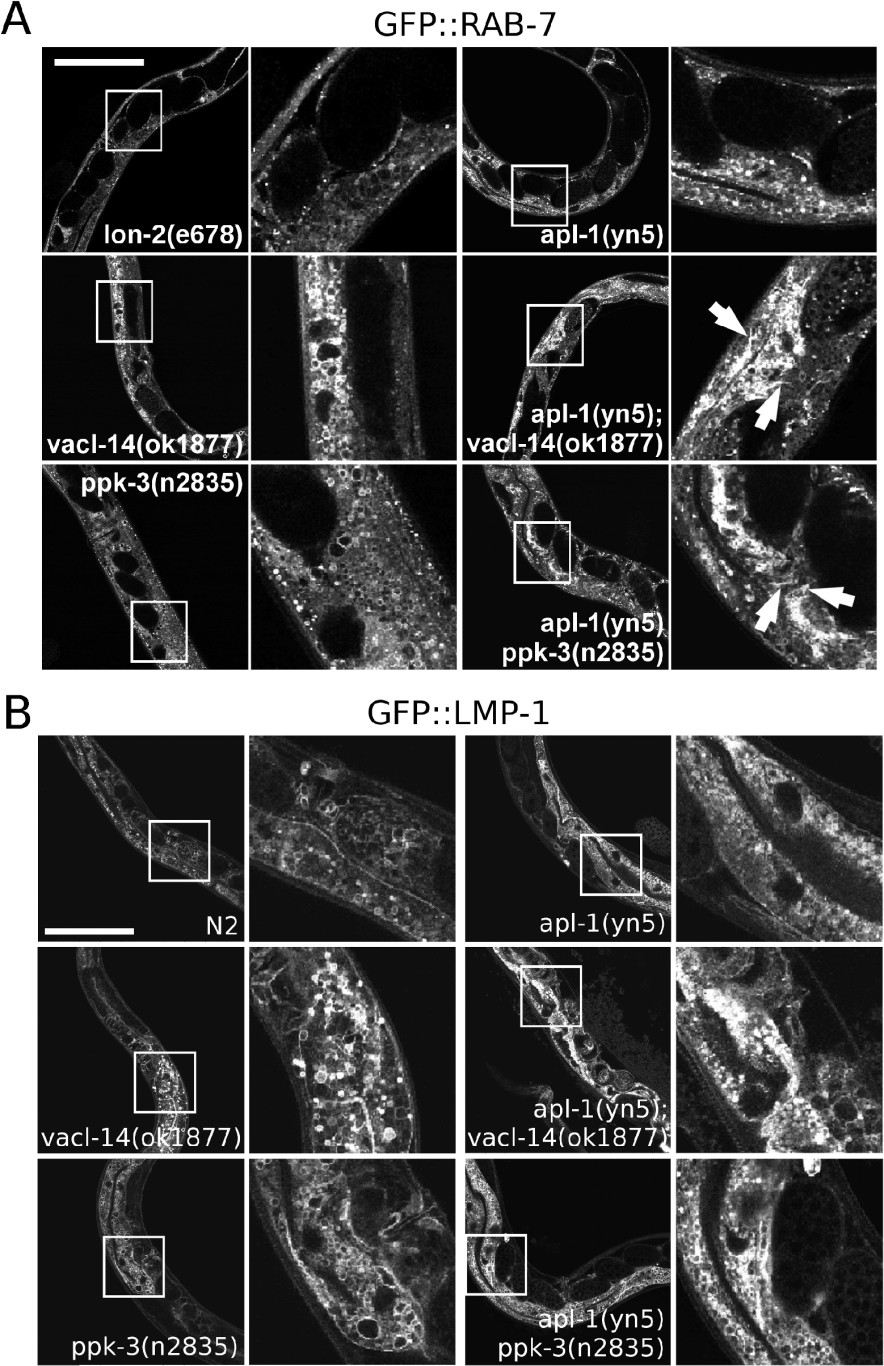
Mutations in apl-1 and ppk-3 or vacl-14 lead to defective processing of late endosomes and lysosomes in young adults. (A) In control animals and single apl-1(yn5), vacl-14(ok1877) and ppk-3(n2835) mutants late endosomes stained using GFP::RAB-7 as a marker were visible as vesicles or small vacuoles. Combination of the mutations in apl-1(yn5); vacl-14(ok1877) or apl-1(yn-5) ppk-3(n2835) double mutants led to a drastic alteration of the morphology of the late endosomal compartment, e.g. late endosomes became tubular and clustered in the hypoderm and intestine when comparing double with single mutants. While in single mutants individual compartments were abundant, they appeared 'squashed' and tubular in the double mutants (indicated by arrows). (B) Analysis of the lysosomal marker GFP::LMP-1 showed that in single apl-1(yn5), vacl-14(ok1877) and ppk-3(n2835) mutants distinct, LMP-1 positive vacuoles of variable sizes were apparent. However, in apl-1(yn5); vacl-14(ok1877) and apl-1(yn5) ppk-3(n2835) double mutants the LMP-1 positive structures appeared aggregated, mimicking the defect observed in GFP::RAB-7 worms, suggesting a defect in late endosomal and lysosomal trafficking. These data showed that APL-1 and the PPK-3 complex are required for late endosomal processing. Bar, 100μm.

The PIKfyve complex is known to regulate several trafficking steps during the maturation of endosomes [15, 24, 34]. As the vacuoles induced by loss of PPK-3 function were shown to be of endosomal/lysosomal origin [15, 18] our data suggest that APL-1 participates in the regulation of endosomal homeostasis through activation of the PPK-3 complex. We therefore characterised the endosomal and lysosomal markers RAB-5, RAB-7 and LMP-1. The early endosomal marker RAB-5 appeared to be largely unaffected by single and double mutations in *apl-1*, *ppk-3* or *vacl-14* (S4 Fig) in agreement with previous data, demonstrating that PIKfyve affects mainly late endosomal compartments [15, 25]. In contrast, the late endosomal and lysosomal markers RAB-7 and LMP-1 were profoundly altered in the *apl-1(yn5) ppk-3(n2835)* and the *apl-1(yn); vacl-14(ok1877)* double mutants and showed late endosomes to be clustered together (Fig 5). These data were corroborated by RNAi suppression of APL-1 in *vacl-14* and *ppk-3* mutants expressing GFP::RAB-7 in which loss of APL-1 by RNAi suppression enhanced late endosomal pathology caused by loss of PPK-3 function (S5 Fig). These defects were observed both in the hypoderm as well as in the intestine. When studying the expression of a promoter fusion of APL-1 with GFP Wiese et al. could not to detect APL-1 expression in the intestine using a 5'-UTR fusion presumed to contain the complete APL-1 promoter [35]. We reassessed the expression pattern of APL-1 using a fosmid from the TransgeneOme project in which a genomic copy of APL-1 was tagged with GFP [36]. We created a transgenic line (OL0186) by microinjection of the APL-1 fosmid followed by analysis of APL-1::GFP expression. Overall the expression pattern was similar to those previously described [32, 35] with APL-1 expression detectable in head muscle cells, head neurons as well as the nerve chords. However, an important difference was that APL-1::GFP expression was also detected in the intestine (S6 Fig). These data indicate that the previously utilised 5'-UTR fusions of APL-1 drive APL-1 expression in most, but not all the tissues compared to a genomic construct. These data suggest that APL-1 is expressed in the *C*. *elegans* intestine in addition to the previously established tissues. This is fully compatible with the intestinal phenotype observed in the double mutants.

In summary these data show that APL-1 regulates late endosomal morphology in concert with the PPK-3 complex.

### APP and PIKfyve control neuronal functions

**Fig 6:**
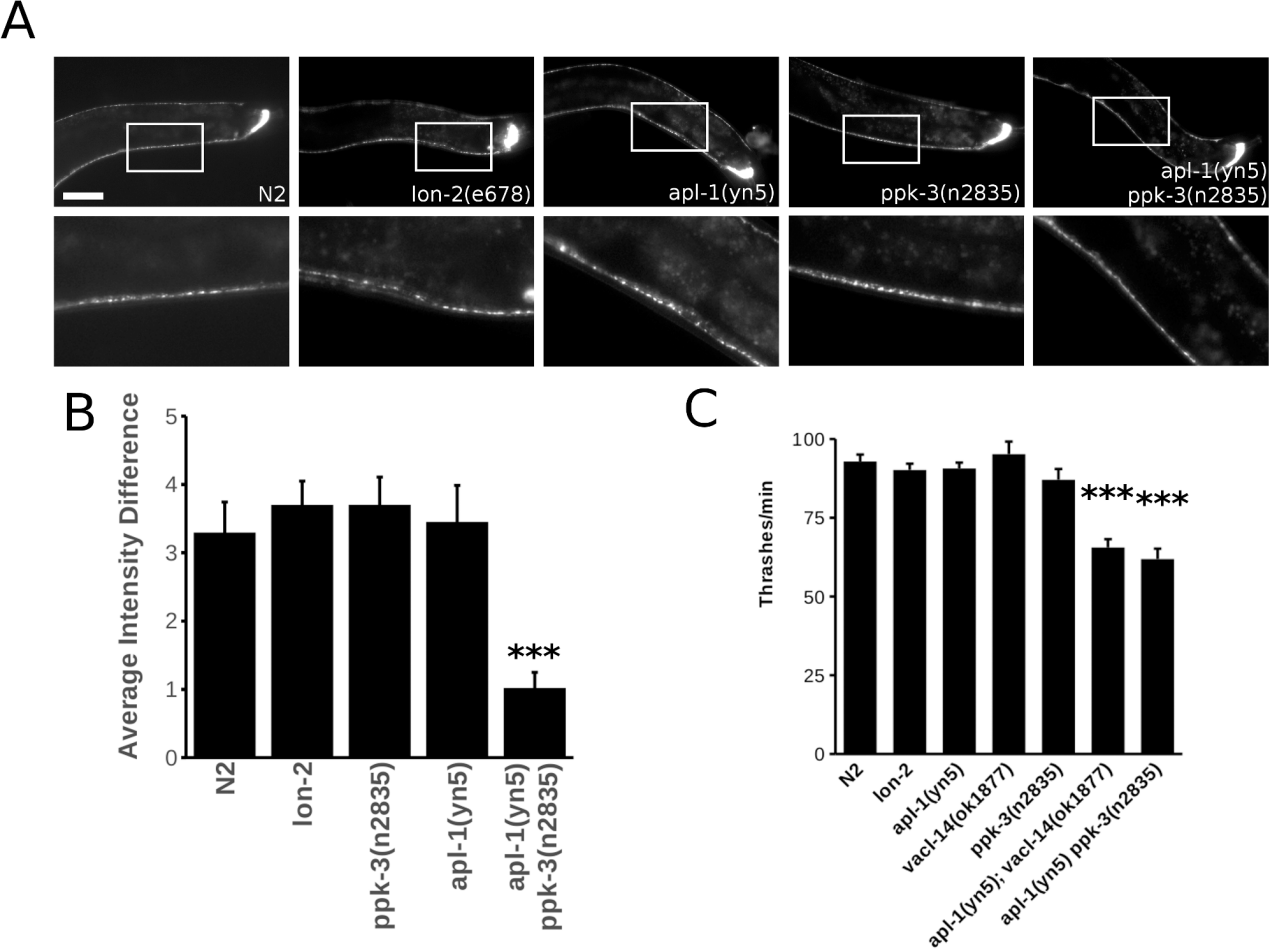
APL-1/PPK-3 interplay is required for neuronal function. (A) The C. elegans synaptic marker GFP::RAB-3 was used to visualise the central nervous system of control animals, single and double mutants. While the nervous system was intact at the light microscopic level in control animals and single mutants, in the apl-1(yn5) ppk-3(n2835) double mutant the accumulation of the synaptic vesicle marker GFP::RAB-3 was impaired leading to a more homogeneous, less well-defined staining of the ventral nerve chord. Enlargement of the respective areas is shown in the second row of panels. Bar, 50 μm. (B) Average intensity difference between GFP::RAB-3 maxima (corresponding to synapses) and GFP::RAB-3 minima (intersynaptic space) measured along the C. elegans ventral nerve chord demonstrated the reduced ability of the apl-1(yn5) ppk-3(n2835) double mutant to concentrate RAB-3 in synapses compared to the N2 control (p<0.01, two-tailed t-test, n>13). Error bars are s.e.m. (C) Thrashing analysis of single and double mutants demonstrated that the interplay between APL-1 and the PPK-3 complex is required for motor control in C. elegans. The double mutants apl-1(yn5); vacl-14(ok1877) and apl-1(yn5) ppk-3(n2835) were significantly impaired in their thrashing compared to the Bristol N2 and lon-2(e678) controls and the single mutants (p values student’s t-test <0.01, n = 20 per strain). Error bars are s.e.m.

As defective PIKfyve function has been shown to cause profound neurodegeneration in mice and in humans [21, 25] and as APP is crucially implicated in Alzheimer's disease we wanted to test whether mutations in *apl-1*, *ppk-3 or vacl-14* can lead to impaired neuronal function in *C*. *elegans*. We characterised the overall morphology of the neuronal system of *C*. *elegans* using the synaptic marker GFP::RAB-3 by fluorescence microscopy (Fig 6). In single *apl-1(yn5)* and *ppk-3(n2835)* mutants the neuronal system appeared to be largely unaffected. However, the *apl-1(yn5) ppk-3(2835)* double mutant failed to accumulate GFP::RAB-3 label in the characteristic “pearls-on-a-string” fashion to the same extent compared to control animals, leading to more homogeneous RAB-3 staining along the ventral nerve chord (Fig 6). Lack of concentrating RAB-3 vesicles in synapses along the ventral nerve chord suggests that synaptic transmission may be impaired.

We wanted to also assess whether the interplay of APL-1 and PPK-3 is functionally important. For this we tested the ability of single and double mutants to coordinate motor function using a thrashing assay. *C*. *elegans* reacts with rapid and sustained thrashing when placed in liquid, a simple assay for testing synaptic transmission of the main motor neurotransmitters, glutamate and gamma amino butyric acid (GABA). While the single mutants *apl-1(yn5)*, *vacl-14(ok1877)* and *ppk-3(n2835)* displayed no significant impairment of motor control, the *apl-1(yn5) ppk-3(n2835)* and *apl-1(yn5); vacl-14(ok1877)* double mutants were both significantly impaired in their ability to thrash (Fig 6). Together with the RAB-3 morphological data this demonstrates that the APL-1/PPK-3 interplay is necessary for supporting neuronal function.

## Discussion

To date the regulation of the PIKfyve complex has remained unclear in multicellular organisms. Our genetic analysis of the interplay between the *C*. *elegans* orthologues of APP and the PIKfyve complex suggests that APP controls the PIKfyve pathway *in-vivo*. This is evidence for a completely novel and unexpected function of APP.

**Fig 7:**
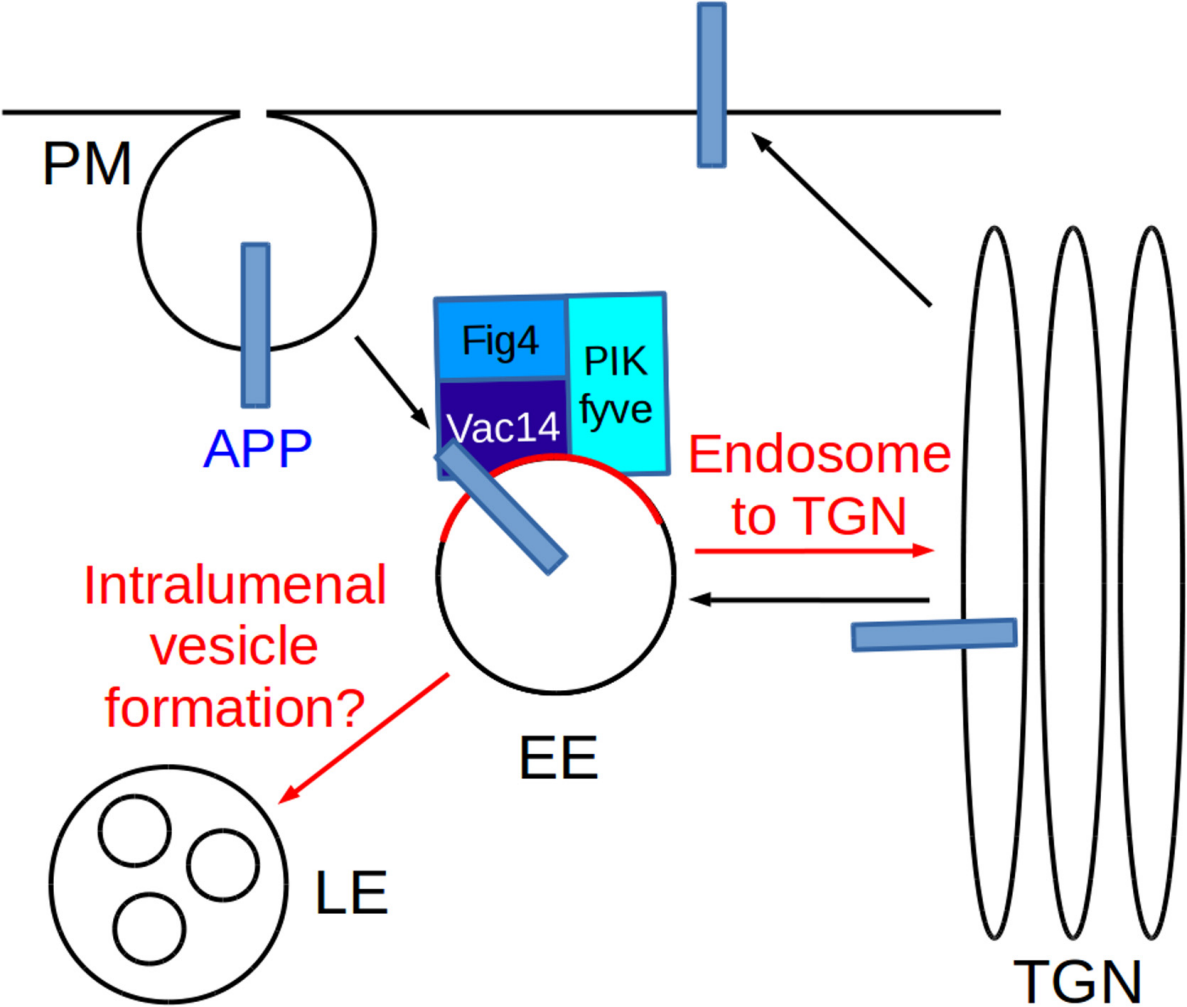
Working model for the interplay of APP with the PIKfyve complex to control endosomal sorting and homeostasis. APP is well known to traffic between the trans-Golgi-network (TGN), the plasma membrane (PM) and the endosomal system consisting of early endosomes (EE), late endosomes (LE) and lysosomes ([10] and references therein). The PIKfyve complex phosphorylates PI(3)P to PI(3,5)P2 (indicated by red labelling of the membrane) which is crucial for endosome-to-TGN transport, endosomal homeostasis [15, 18, 25] and other endosomal sorting processes during the maturation of endosomes [47]. In this study we have shown that APP can associate via its intracellular domain with Vac14 of the PIKfyve complex and that the C. elegans APP orthologue APL-1 functionally cooperates with the PIKfyve/PPK-3 complex in C. elegans. We propose a working model in which APP/APL-1 traffics through the vesicular transport system and, upon arrival in endosomes, interacts with the PIKfyve/PPK-3 complex via its intracellular domain. The genetic data obtained in C. elegans suggest that APL-1 stimulates PPK-3 activity which is necessary to maintain endosomal function. Compromised PIKfyve/PPK-3 function impacts on endosomal sorting and homeostasis which in C. elegans as well as in mammals has detrimental consequences for neuronal function and integrity [17, 25, 26].

As a working model we would like to propose that APP, upon arrival to endosomes, interacts with and activates the PIKfyve complex to stimulate processes such as endosome-to-TGN transport and endosome/lysosome fusion (Fig 7). Previous work in multiple systems has established that APP/APL-1 traffics through the endosomal system, which in *C*. *elegans* is required for maintenance of APL-1 [35]. The system we describe here would link APP trafficking to the production of the crucial endosomal signalling lipid PI(3,5)P_2_ and potentially PI(5)P [17].

One of the many gaps in our understanding of the PIKfyve/PI(3,5)P_2_ system is through which effectors PI(3,5)P_2_ mediates its effects. In mammals one of the few established effectors is the endo/lysosomal, Ca^2+^-selective TRPML1 channel, also known as mucolipin [18]. Dong and colleagues demonstrated that drop of PI(3,5)P_2_ levels caused by PIKfyve deficiency prevents activation of TRPML1, thus blocking Ca^2+^ release from the lumen of endosomes. This release is required for endo/lysosomal fusion events. Impaired lysosomal fusion blocks the flux through the endosomal system leading to endosomal swelling [18]. Interestingly, mutations in mucolipin lead to lysosomal storage disease and neurodegeneration [19].

While it is clear that APP's processing is intimately linked with Alzheimer's disease there is currently no clear mechanistic model for the molecular events that lead to the induction and progression of this disease. For the last two decades the amyloid cascade hypothesis has dominated the field. Its core assumption is that production of beta amyloid initiates a cascade that ultimately leads to 'plaque' deposition in patients' brains, formation of neurofibrillar tangles and neuronal death resulting in neurodegeneration [37]. However, immunological approaches of targeting beta amyloid production or deposition have essentially failed to stop cognitive decline and neurodegeneration [38]. This raises the possibility that progression of Alzheimer's disease is driven by processes other than beta amyloid accumulation [39, 40]. It is conceivable that loss-of-APP function rather than the toxic gain-of-function of beta amyloid drives neurodegeneration in Alzheimer's disease. However, as the cellular function of APP remained unclear, describing how loss of APP function could contribute to Alzheimer’s disease was not possible to date [41]. Our finding that APP regulates the PIKfyve complex in *C*. *elegans* in vivo hints at a novel molecular mechanism for neurodegeneration in Alzheimer's disease in which aberrant processing of APP by beta and gamma secretases would preclude APP from binding to and activating the PIKfyve complex. This would lead to a drop of endosomal PI(3,5)P_2_ level which would disrupt endosomal sorting and homeostasis through a lack of activation of PI(3,5)P_2_ effectors, the TRPML1 channel mucolipin being one of them [18]. Loss of PIKfyve subunits and TRPML1 are well known to result in neurodegeneration. This idea would also account for the aberrant accumulation of vesicles with endo- and lysosomal characteristics observed in the brains of Alzheimer's patients (reviewed in [42]). Damage to the endo/lysosomal system of neuronal cells will impact on the clearance of beta amyloid by endocytosis and lysosomal degradation, potentially explaining the accumulation of beta amyloid and the resulting pathological effects of this molecule.

Exploring this idea will yield fascinating insights into the regulation and significance of the PI(3,5)P_2_ metabolism and may redefine our view of Alzheimer’s disease.

## Materials and Methods

### Antibodies

The antibody directed against Vac14 was kindly provided by Prof. L. Weisman (University of Michigan). Antibodies against EEA1 were purchased from BD Biosciences and Lampl from Santa Cruz Biotechnology.

### Proteo-liposomes

Protein recruitments and mass spectrometry were carried out as established in [27] with the following modifications: 0.5ml of mouse brain cytosol (final concentration 3mg/ml) were used supplemented with 0.21mM GTPγS and 28μM latrunculin B. The xcalibur.raw files were processed with MaxQuant and the Perseus software of this package was used for analysis as described in [30]. Briefly, LFQ intensities were logarithmised and only proteins that were identified in all replicates of at least one of the samples were retained. Missing values were imputed by a normal distribution around the detection limit and a modified t-test (SAM) was performed with a threshold value of 0.05 and a slope value of 1. A detailed description of the method is presented in [43].

### *C*. *elegans* methods and strains

Worm maintenance, genetic crosses and other *C*. *elegans* methods were performed according to standard protocols [44]. All strains were grown at 20°C. Mutations used were LGI, *vacl-14(ok1877)*; LGX, *apl-1(yn5) lon-2(e678)*, lon-2(e678), *ppk-3(n2835)*, *ppk-3(n2668) *[*33]. vacl-14(ok1877)* was back-crossed three times to WT strain N2 prior to further analysis. The *ok1877* deletion was followed by PCR. As negative controls either Bristol N2 wildtype or *lon-2(e678)* were used as the *apl-1(yn5)* mutation is in close proximity to the *lon-2* locus which served as a marker to follow the *yn5* deletion throughout crosses.

Strains carrying integrated arrays used in this study were: *pwIs50 (*p*lmp-1*::*LMP-1*::*GFP)*, *pwIs439 (*p*rab-5*::*GFP*::*RAB-5)*, *pwIs399 [*p*rab-7*::GFP::RAB-7) were kindly provided by Prof. B.Grant, *jsIs682 [*p*rab-3*::*GFP*::*RAB-3)* by CGC. For RNA interference (RNAi) experiments, *apl-1* cDNA was prepared from EST clone provided by Prof. Kohara (National Institute of Genetics, Japan) and subcloned into RNAi feeding vector L4440 [45]. RNA interference (RNAi) was performed by the feeding method [45]. For RNAi knockdown of *apl-1*, L1 worms were placed onto RNAi plates, and P0 adults were scored for phenotype after 72 h.

### Transgenic strain

The transgenic APL-1::GFP strain was produced as previously described [46]. Briefly, N2 animals were bleached and synchronised. Young adults were picked with an eyelash and placed on an injection pad (2% agarose in M9 buffer). Worms were coinjected with 50 ng/μl *apl-1* fosmid (Construct: 10208337314574834 H09, obtained from TransgeneOme) and 120 ng/μl pRF4 roller plasmid as coinjection marker. 20–30 worms were injected and analysed for GFP positive offspring. GFP positive offspring was isolated to create line OL0186.

### Vacuole number and size

was detected using ImageJ by manual circling each individual vacuole present in a DIC image of the anterior tip of the worm and measuring number and size of them using the “Analyse Particle” command.

### GFP::RAB-3 accumulation

Using ImageJ the intensity profile of GFP::RAB-3 was established along the ventral nerve chord using manually created line plots, maximas and minimas detected and the average intensity difference between maxima and minima were calculated.

### Thrashing assay

L4 larval stage animals were picked onto fresh OP50 seeded plates and scored for thrashing as young adults 24 hours later. Individual animals were transferred into 30μl drop of M9 buffer into a depression well slide and thrashing was scored for 1 minutes after worms were allowed to settle for 1 minute. A single thrash was defined as a complete change in the direction of the body down the midline.

### Microscopy

Fluorescence and DIC images were obtained using a fully motorized Zeiss Axiovert 200M fluorescence microscope (Carl Zeiss) and a Hamamatsu Orca camera controlled by Volocity software (Improvision). Confocal images were obtained using a Leica SP5 upright confocal microscopy setup. To observe live worms expressing transgenes, worms were mounted on 2% agarose pads containing 0.1M tetramisole in M9 buffer.

### Vesicle tracking

was performed using the 'Manual Tracking' plugin in ImageJ.

### Intensity quantification of RAB-3 staining

Quantification was performed using ImageJ. Briefly, a line was manually drawn along the ventral nerve chords and the pixel intensity were measured in a line plot. Intensity maxima and minima were defined and the average difference between the two was measured.

## Supporting Information

S1 Fig Line scans in HeLa cells expressing APP-CFP and Vac14-mCIT highlight colocalisation between APP and Vac14. (TIF)

S2 Fig Truncation of APL-1 in *ppk-3* or *vacl-14* mutants significantly enhanced the vacuolar phenotype. DIC images were collected from the vulval area of young control adults (*Bristol N2*), single and double mutants. Examples of vacuoles are labelled with arrows, asterisks indicate very large vacuoles. The *apl-1(yn5) ppk-3(n2835)* and *apl-1(yn5); vacl-14(ok1877)* displayed significantly larger and more vacuoles than the single mutants or wildtype controls. Note that the *apl-1(yn5) ppk-3(n2668)* were heavily vacuolated and did not produce viable offspring. Bar, 50μm.
(TIF)

S3 Fig RNAi suppression of APL-1 in control animals and *ppk-3* mutants enhances the vacuolar phenotype caused by loss of PPK-3 activity. The mutants indicated in the left column were fed with a bacterial strain containing a control RNAi-plasmid (L4440), in the right column a strain containing an APL-1 targeting RNAi plasmid. Arrows indicate vacuoles in hypodermal cells. Asterisks indicate very large vacuoles.
(TIF)

S4 Fig Characterisation of early endosomes in APL-1 and PPK-3 mutants. The early endosomal, RAB-5 positive compartment remained largely unaffected by mutations in *apl-1* and the PPK-3 complex. Bar, 100μm.
(TIF)

S5 Fig RNAi suppression of APL-1 in animals with impaired PPK-3 function enhanced late endosomal defects as visualised by GFP::RAB-7. Suppression of APL-1 (right column) altered the morphology of late endosomes with mutations in *ppk-3* and *vacl-14* compared with the control RNAi L4440 (left). Late endosomes appeared to either aggregate, swell in size (indicated by arrows) or undergo tubulation with the effects most visible in hypodermal cells. Bar, 50μm.
(TIF)

S6 Fig APL-1 expression pattern of N2 transfected with a fosmid encoding a genomic copy of APL-1 tagged with GFP. APL-1::GFP expression is detected in neuronal and non-neuronal cells, amongst them head muscle cells (A), the nerve chord (indicated by arrow) (B), the hypoderm (C) and the intestine (D). DIC images are indicated by'. Bar, 50μm.
(JPG)

S1 Video Colocalisation and comigration of vesicles labelled with APP-mCherry (red) and Vac14-mCit (green) in transiently transfected HeLa cells. Speed ~8x.
(AVI)

S2 Video Colocalisation and comigration of vesicles labelled with AICD-mCherry (red) and Vac14-mCit (green) in transiently transfected HeLa cells. Speed ~8x.
(AVI)
