# Supplementary figures and images for: The Amyloid Precursor Protein Controls PIKfyve Function
Source: PLoS One. 2015 Jun 30;10(6):e0130485. doi: 10.1371/journal.pone.0130485 (PMC4488396; doi:10.1371/journal.pone.0130485)

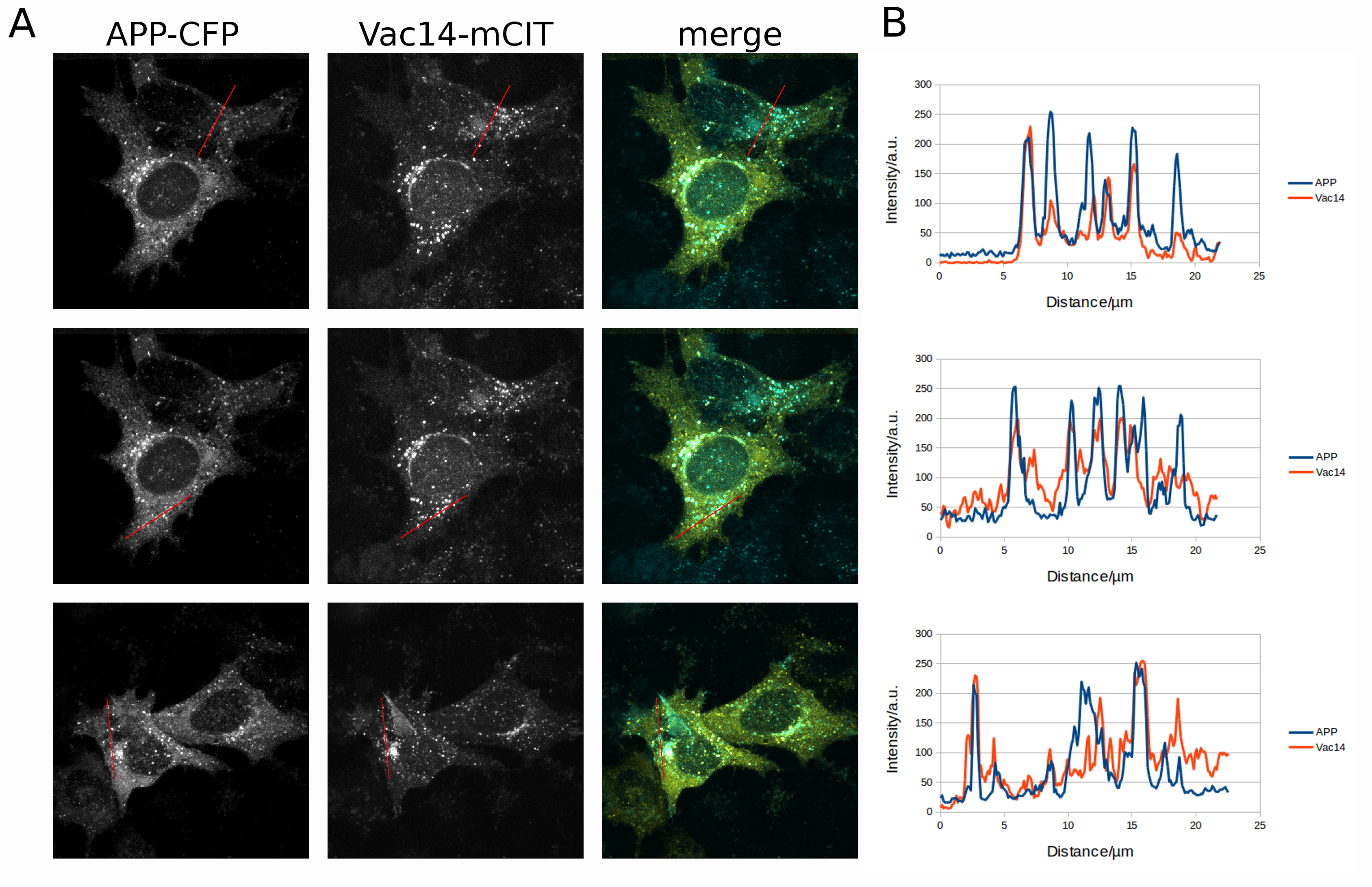

Supplement: S1 Fig — (TIF) [file pone.0130485.s001.tif]

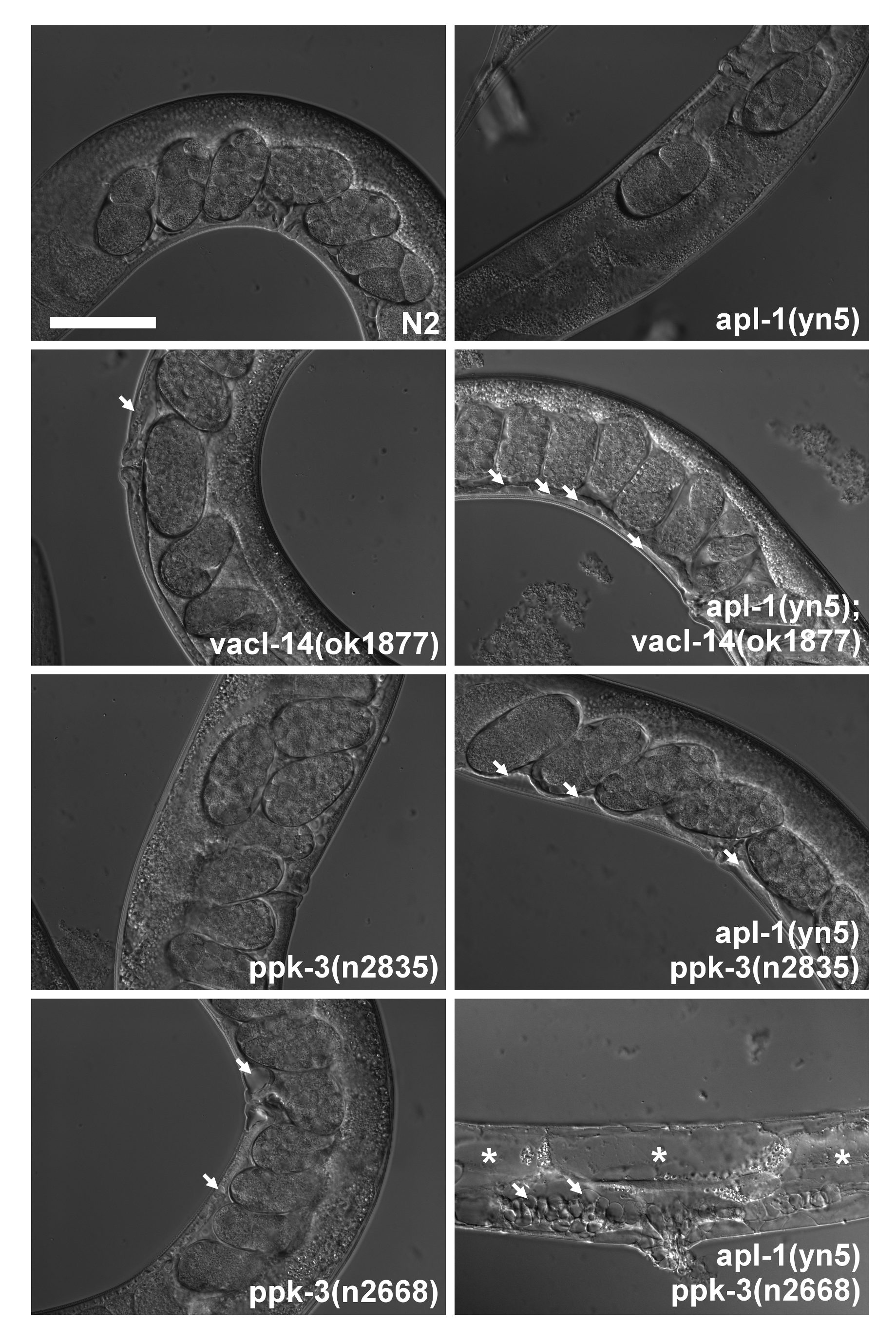

Supplement: S2 Fig — DIC images were collected from the vulval area of young control adults (Bristol N2), single and double mutants. Examples of vacuoles are labelled with arrows, asterisks indicate very large vacuoles. The apl-1(yn5) ppk-3(n2835) and apl-1(yn5); vacl-14(ok1877) displayed significantly larger and more vacuoles than the single mutants or wildtype controls. Note that the apl-1(yn5) ppk-3(n2668) were heavily vacuolated and did not produce viable offspring. Bar, 50μm. (TIF) [file pone.0130485.s002.tif]

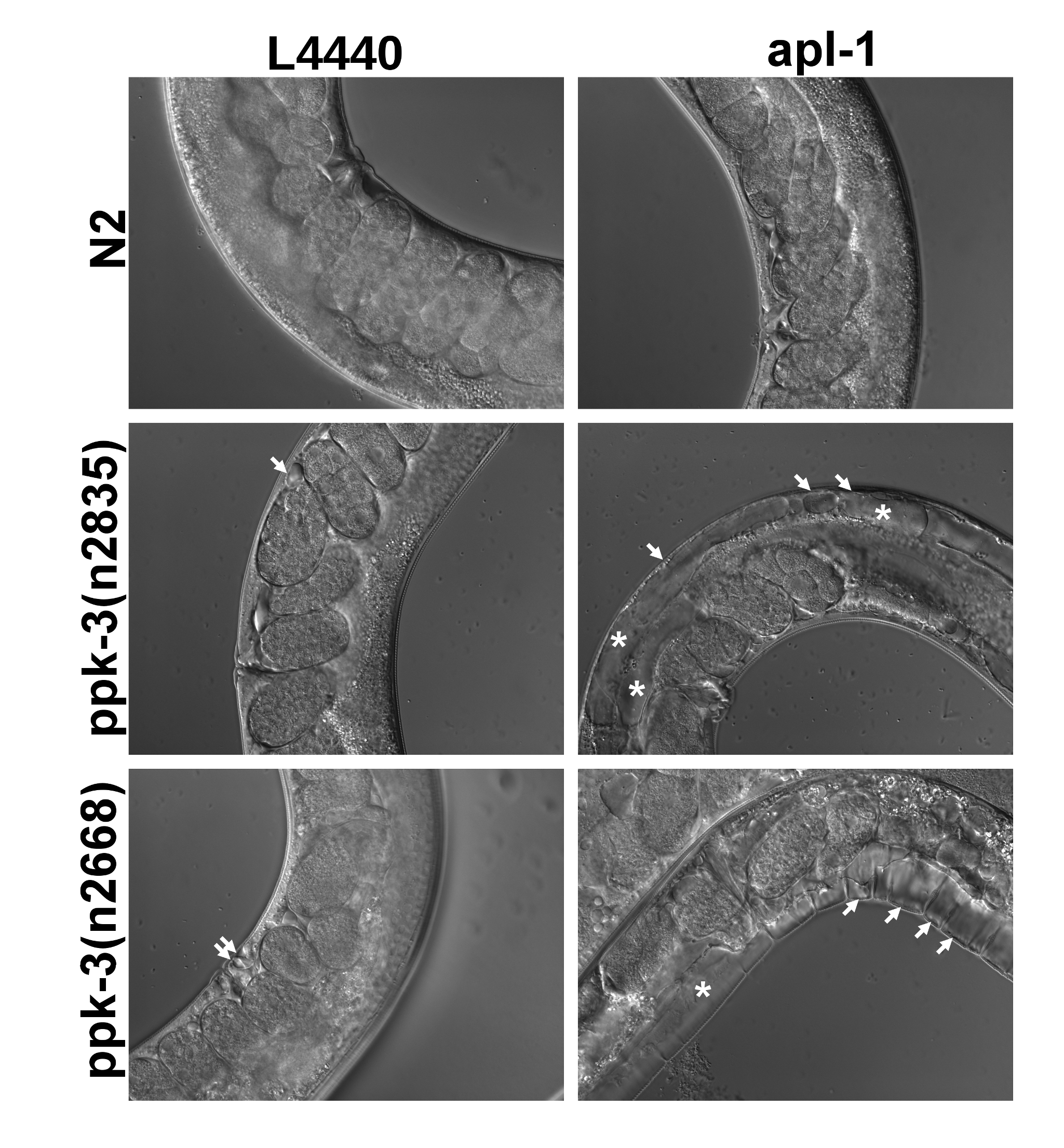

Supplement: S3 Fig — The mutants indicated in the left column were fed with a bacterial strain containing a control RNAi-plasmid (L4440), in the right column a strain containing an APL-1 targeting RNAi plasmid. Arrows indicate vacuoles in hypodermal cells. Asterisks indicate very large vacuoles. (TIF) [file pone.0130485.s003.tif]

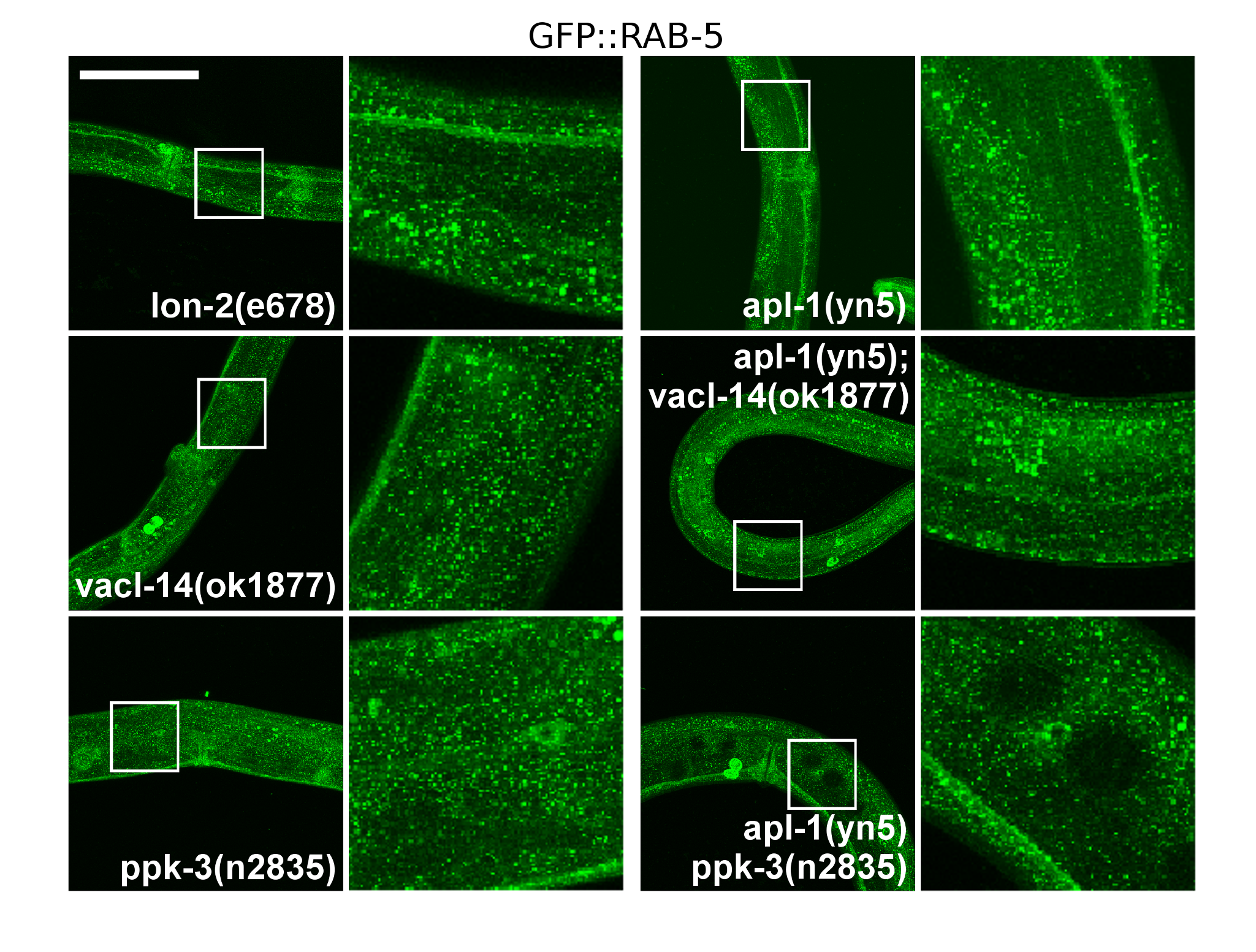

Supplement: S4 Fig — The early endosomal, RAB-5 positive compartment remained largely unaffected by mutations in apl-1 and the PPK-3 complex. Bar, 100μm. (TIF) [file pone.0130485.s004.tif]

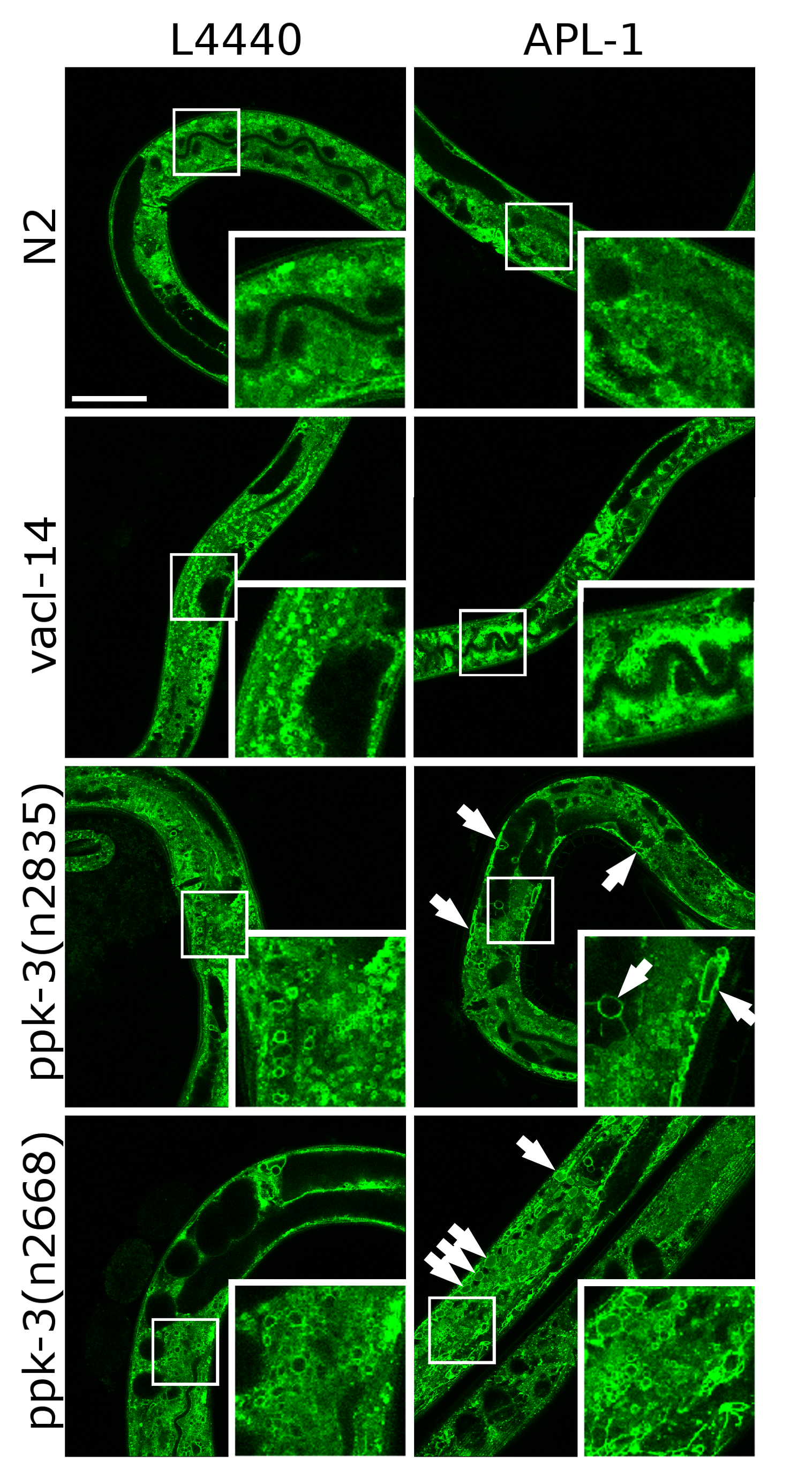

Supplement: S5 Fig — Suppression of APL-1 (right column) altered the morphology of late endosomes with mutations in ppk-3 and vacl-14 compared with the control RNAi L4440 (left). Late endosomes appeared to either aggregate, swell in size (indicated by arrows) or undergo tubulation with the effects most visible in hypodermal cells. Bar, 50μm. (TIF) [file pone.0130485.s005.tif]

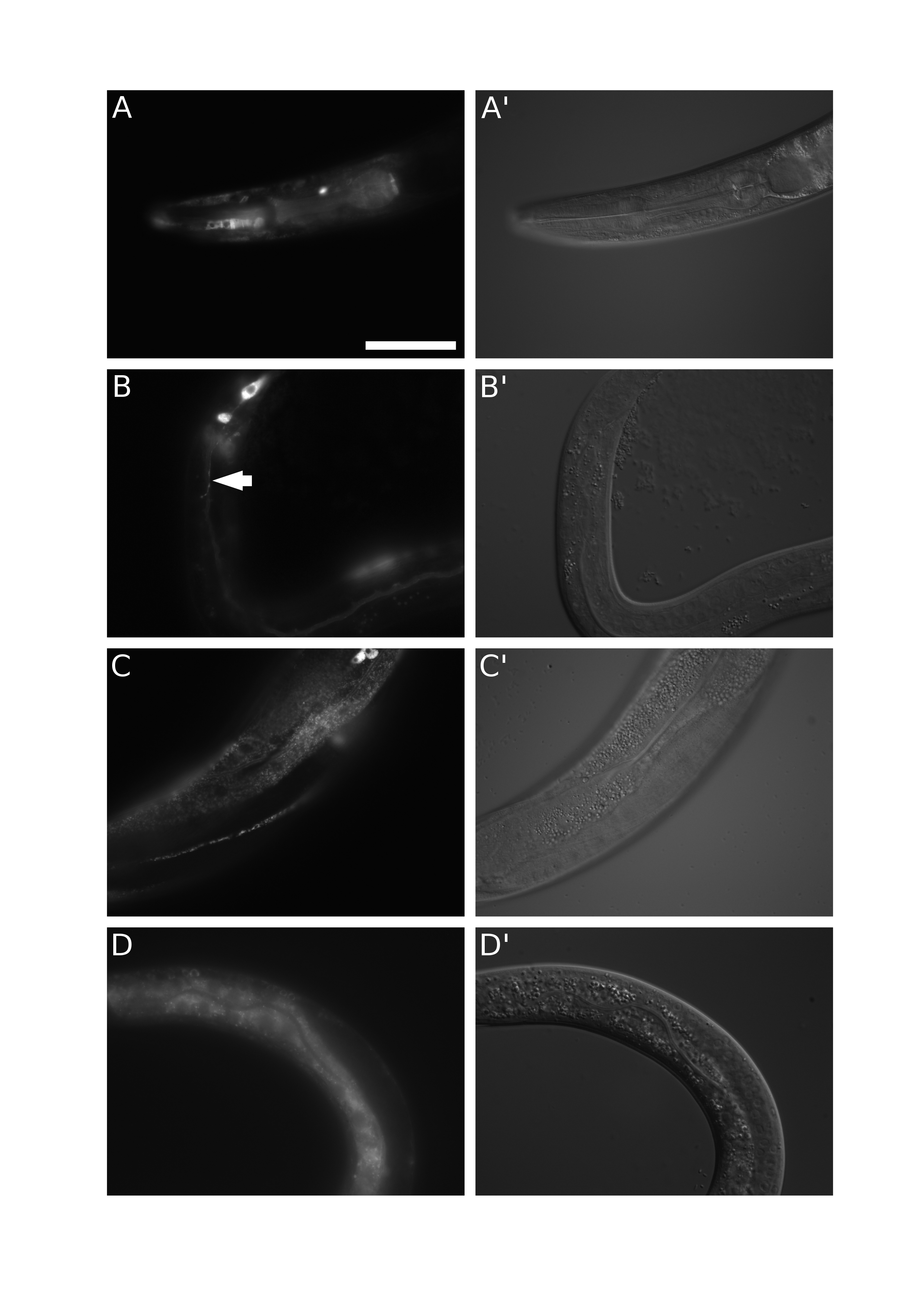

Supplement: S6 Fig — APL-1::GFP expression is detected in neuronal and non-neuronal cells, amongst them head muscle cells (A), the nerve chord (indicated by arrow) (B), the hypoderm (C) and the intestine (D). DIC images are indicated by'. Bar, 50μm. (JPG) [file pone.0130485.s006.jpg]
